# Insect Cell-Based Models: Cell Line Establishment and Application in Insecticide Screening and Toxicology Research

**DOI:** 10.3390/insects14020104

**Published:** 2023-01-18

**Authors:** Xuan He, Lidan Lu, Ping Huang, Bo Yu, Lianxin Peng, Liang Zou, Yuanhang Ren

**Affiliations:** Key Laboratory of Coarse Cereal Processing, Ministry of Agriculture and Rural Affairs, Sichuan Engineering and Technology Research Center of Coarse Cereal Industralization, College of Food and Biological Engineering, Chengdu University, Chengdu 610106, China

**Keywords:** insect, cell lines, insecticide, high-throughput screening, mode of action

## Abstract

**Simple Summary:**

Insect cell lines have often been employed in pest management, where they have been used as tools to evaluate the activity and explore the toxic mechanisms of insecticide candidate compounds. This review summarizes the progression of insect cell line establishment, then introduces several recent studies based on insect cell lines coupled with advanced technologies. These cell-based studies could increase efficiency and reduce the cost of insecticide studies, providing a global and in-depth perspective to reveal and verify the mode of action of insecticides.

**Abstract:**

During the past decades, research on insect cell culture has grown tremendously. Thousands of lines have been established from different species of insect orders, originating from several tissue sources. These cell lines have often been employed in insect science research. In particular, they have played important roles in pest management, where they have been used as tools to evaluate the activity and explore the toxic mechanisms of insecticide candidate compounds. This review intends to first briefly summarize the progression of insect cell line establishment. Then, several recent studies based on insect cell lines coupled with advanced technologies are introduced. These investigations revealed that insect cell lines can be exploited as novel models with unique advantages such as increased efficiency and reduced cost compared with traditional insecticide research. Most notably, the insect cell line-based models provide a global and in-depth perspective to study the toxicology mechanisms of insecticides. However, challenges and limitations still exist, especially in the connection between in vitro activity and in vivo effectiveness. Despite all this, recent advances have suggested that insect cell line-based models promote the progress and sensible application of insecticides, which benefits pest management.

## 1. Introduction

The consistent increase in the global population has highlighted the need to improve the quantity and quality of food production. Insecticides are one of the most effective tools that people have ever invented to control pests. According to statistics, insecticides can prevent crop reduction by 21 to 94% in a given year [1]. Hence, the development of new insecticides to control pests has been a critical need for centuries that continues today. However, the discovery and commercialization of a new crop protection compound is a huge and complex systematic undertaking and has become progressively expensive. As the most important part of such a job, the evaluation of insecticidal activity and the study of the mode of action of candidates are time-consuming and costly, especially when using insect and mammal models in vivo as experimental subjects. Therefore, establishing and developing certain new models for substituting or complementing the traditional model is becoming a feasible approach to reducing costs and improving efficiency.

Improvements in modern life sciences technologies, such as molecular and cell biology, have facilitated the in-depth analysis of effects on the insect body by focusing on cells. During the past decades, many studies have been performed to establish insect cell lines. Thousands of lines have been established from different species of insect orders, originating from several tissue sources. Aside from their notable role in protein expression, the insect cell lines have been applied in a wide area of study, especially as screening models for candidate compound discovery in pest management. These insect cell lines also have been employed extensively for the mechanism of insecticidal chemical substances in vitro. Insect cell-based models have been proven to be advantageous for activity evaluation not only owing to their low cost/benefit ratio, ease of maintenance, repeatability and opportunity for use in high-throughput screening, but also because their use does not raise bioethical conundrums. As a supplement and expansion of traditional toxicological study models, insect cell-based models could further provide a comprehensive and in-depth perspective to study the toxicology of insecticides.

The aim of this review is to summarize the establishment of insect cell lines and introduce the application of insect cell-based models in the field of insecticide research and development as high-throughput screening models for the biological activity assessment of synthetic compounds and purification of natural products, and as tools for determining the molecular mechanisms of insecticides from the perspective of cell death signaling pathways, ion channels and omics. Then, major advantages and challenges for insect cell-based models are discussed.

## 2. Establishment of Insect Cell Lines

### 2.1. Initiation and Progression of Insect Cell Lines

The first insect cell line was generally acknowledged to be derived from the *Antherea eucalypti* by Grace in 1962. However, in fact, Chinese researchers obtained an insect cell line originating from silk moths earlier than Grace [2]. Although the first insect cell line was derived from Lepidoptera, primary efforts to establish insect cell lines were focused on mosquitos. The aim was to separate and describe arboviruses that cause serious diseases in humans. Success was not observed until the first report of the cell line from *Aedes aegypti* was published in 1966 [3]. A few years later, cell line establishment efforts changed to Culex mosquitoes because they are vectors of critical arbovirus diseases [4]. Researchers successfully established a cell line from *Culex bitaeniorhynchus* and it was employed primarily to study the Japanese encephalitis virus in vitro [5]. In addition, cell lines derived from Lepidoptera are numerous because they are also hosts for many viruses. The most-applied cell lines for the replication of Autographa californica nucleopolyhedrovirus (AcMNPV) were derived from a kind of fall army worm *Spodoptera frugiperda*, [6]. Sf21 originated from *S. frugiperda*, and its derivative, Sf9, have been used widely in AcMNPV replication, exogenous protein expression [7] and vaccine production [8]. Then, a new cell line originally derived from *Trichoplusia ni* embryos was found to be highly susceptible to AcMNPV. This was named BTI-Tn5b1-4 or “Hi-Five”, due to its high performance in recombinant protein production [9,10]. As a very important research tool, research on insect cell lines has also developed rapidly in recent years. For example, Watanabe [11] built novel cell lines originating from the fat bodies of six lepidopteran insects. Zhang [12] reported the creation of cell lines from the fat body, nervous system and reproductive tissue of *Spodoptera eridania* larvae. Zhou [13] reported three established cell lines, DvWL1, 2 and 3, which were first derived from *Diabrotica virgifera virgifera*.

With progress in aseptic technology, microbial contamination, which is the main problem in the early stages of cell line establishment, has been solved well. Thereafter, researchers acknowledged that the culture medium is of critical importance in propagating and maintaining cells [14]. Culture media are complex, consisting of carbohydrates, salts, amino acids, growth factors, vitamins, metabolic precursors, hormones and trace elements. The constituents and proportions for these requirements differ for various cell lines. Glucose is generally used as the carbohydrate, but, in a few cases, galactose is supplied to reduce lactic acid aggregation in the medium. Other carbon sources such as L-glutamine and pyruvate are also added into media as supplements. It is noteworthy that serum is important part of a complete medium; fetal bovine serum (FBS) is a good additive for insect cell culture, and a concentration of 5–20% FBS is widely used in insect cell culture. Sodium bicarbonate, phosphate or more buffering systems such as 2-[4-(2-hydroxyethyl)piperazin-1-yl]ethanesulfonic acid (Hepes) are often used to maintain pH. Benefiting from aseptic techniques and the optimization of culture media, insect cell line establishment has grown rapidly. To date, more than one thousand insect cell lines have been successfully established (www.cellosaurus.org/, accessed on 26 October 2022).

### 2.2. Source of Insect Cell Lines

**Figure 1:**
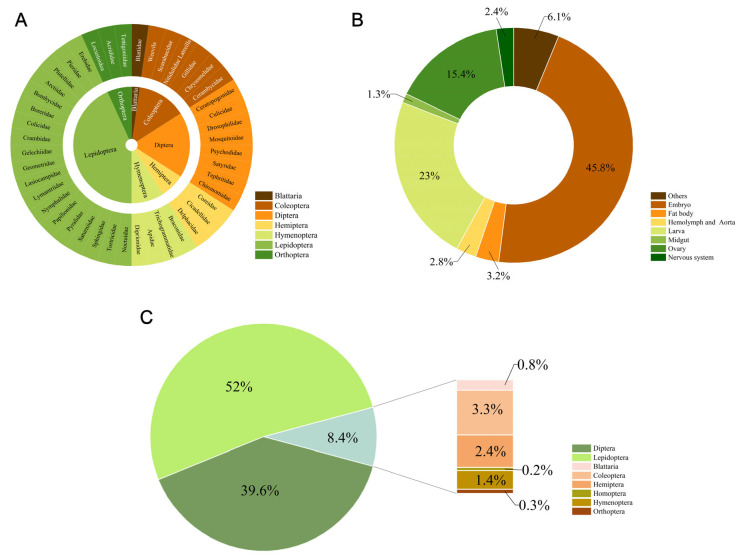
The statistics of established insect cell lines. (**A**) The main order and family source of current established insect cell lines. (**B**) The proportion of different tissues contributing to established insect cell lines. (**C**) The proportion of different orders contributing to established insect cell lines.

There are more than one million species of insects living on Earth [15], but, thus far, according to Expasy-Cellosaurus records, there are only approximately 1200 cell lines from more than 170 distinct species of eight orders (Blattaria, Coleoptera, Diptera, Hymenoptera, Homoptera, Hemiptera, Lepidoptera and Orthoptera) [16]. As shown in Figure 1, more than half of insect cell lines have been derived from Lepidoptera, the major category of pests against crops. Lepidopteran insects have contributed more than 600 reported cell lines originating from Noctuidae, Tortricidae, Sphingidae and 16 other families. Diptera is the second largest source of insect cell lines, with dipteran cell lines comprising approximately 40 percent of the total number. To date, a few cell lines have been reported to originate from Coleoptera, Blattaria, Hymenoptera, Homoptera, Orthoptera and Hemiptera. Among them, the cell lines derived from Homoptera are the fewest.

From the tissue perspective, insect cell lines are isolated from different tissues and organs at different stages of development. One of the hallmarks in insect tissue culture was the first establishment of the cell line from embryos of *T. ni* [17]. By and large, most insect cell lines have been developed from embryos, neonate larvae and ovaries (Figure 1B). Compared with isolated organs of insects used in primary culture, undifferentiated embryonic tissues appeared to be the favorite starting material, leading to more cell lines than any other tissue. According to the statistics, nearly half of insect cell lines were derived from embryonic tissues, followed by larvae (23%) and ovaries (15.4%). Over the past years, many insect cell lines that are initiated from specialized tissues such as the central nervous system and midgut have been developed. Studies on the insect nervous system and midgut are highly important for the understanding of targets, absorption and metabolizing of pesticides, as well as for pesticide discovery.

Many cell lines derived from midgut tissues have been already built from several insect species. They include BCIRL-HzMG8 [18] and RP-HzGUT-AW1 [19], two lines from *Helicoverpa zea*, BPH22 from *Poekilocerus pictus* [20], BTI-TnMG1 from *T. ni* [21] and, more recently, the two lines SfMG1-0611 and SfMG-0617 from *S. frugiperda* [22]. Different midgut cell lines show variable characteristics compared in vivo, including variable responsiveness to Bt Cry proteins or virus infection.

Compared to the midgut, neurons are highly differentiated cells. Hence, it was considered very difficult to establish cell lines from the insect nervous system until Kumiko [23] reported that eight continuous cell lines had been established from the central nervous system of 3rd *Drosophila melanogaster* instar larvae. These cell lines can react to a specific antibody for a neuronal marker of insects and were determined to contain acetylcholine, a neurotransmitter known in *Drosophila*, using high-performance liquid chromatography. After that, Goodman [19] outlined the creation of new cell cultures from primarily neural tissue (larval ventral nerve cords) of *Heliothis virescens* and *H. zea* and their initial characterization. Currently, because they work rapidly and are highly efficient, insecticides that target the nervous system of pests have become the main category of agricultural chemicals and account for a large market share. Hence, the establishment of insect nerve cell lines provides new tools and angles to evaluate toxicity and reveal mechanisms of nerve insecticides at the cellular level.

### 2.3. Verification of Insect Cell Lines

In order to verify the origin and function of insect cell lines, a series of biochemical assays including Western blot, radiolabeled ligands, immunofluorescence and molecular techniques are used by. Goodman [19] generated a number of cell lines from a variety of insect tissues, including four lines from larval ventral nerve cords, one line from larval midguts, one line from adult ovaries and 11 lines from embryonic tissues. These cell lines were primarily characterized through morphological examination. They consisted of cells with varied morphologies, ranging from spherical to elongated, and included ventral nerve cord lines that appeared to form networks in culture. Additionally, these established cell lines were subjected to two different methods of polymerase chain reaction analysis (PCR) for identification purposes. ISSR-PCR results showed that using a variety of primers could distinguish between cell lines from different species. Another case regarded nerve cells as well. The IPLB-CPB2 cell line was derived from eggs of the *Leptinotarsa decimlineata* (Colorado potato beetle). The cell line displayed certain neural-like properties via immunoreactivities. Indirect immunofluorescent results showed IPLB-CPB2 express neurofilament (Nf)-like immunoreactivities to antibodies directed against mammalian Nf-Medium and a heavily phosphorylated form of Nf-Heavy. In addition, the outcomes indicated that the cells express an antigenic epitope characteristic of the mammalian type 1 inositol irisphosphate receptor, the ryanodine receptor and the sarco (endo) plasmic reticulum Ca^2+^ pump. Patch-clamp recordings revealed that part of IPLB-CPB2 cells were capable of producing spontaneous action potentials. The study suggested that IPLB-CPB2 cells were of neural origin with neural-like properties, which could be an excellent model for insect neurobiology and calcium-based signal transduction studies [24].

## 3. Application of Insect Cell Lines in Pest Management

### 3.1. Common Insect Cell Lines in Pest Management

The *D. melanogaster* has been broadly applied in genetic biology. As a model organism, the S2 cell line derived from *D. melanogaster* has also played a vital role in pest management [25]. *Bombyx mori* as the typical representative of Lepidoptera, the silkworm hemocytes and BmN cell line have become important models in the study of apoptosis and insecticide resistance [26,27]. Sf21 and Sf9 cells originating from *S. frugiperda* and SL-1 cells obtained from *Spodoptera litura* have become the basic materials to study toxic effects and the signal pathway of programmed cell death (PCD) [28,29,30]. Moreover, the TN-5B1-4 cell line from *T. ni*, the Hz cell line from *Heliothis Zea* and the Spex cell line from *Spodoptera exigua* are all frequently used models for research on pesticide mechanisms [31,32,33]. The AW1 cell line obtained from the ventral nerve cord of *H. zea* larvae is a specific model for screening and revealing the mechanisms of neuroactive insecticides [34]. In the future, with isolation and culture techniques developing, there might be more insect cell lines obtained from specific organisms and tissues for particular mechanisms’ study.

### 3.2. High-Throughput Screening (HTS)

In order to achieve screening aims, there is increasing concern around developing in vitro methods to substitute traditional animal activity assays. Despite activity tests on individual insects being more dependable, there are some restrictive conditions, such as the fact that a large number of insects with similar physiological conditions are needed, and it requires a long testing period, which leads to time extensions and labor increases. Considering the rapid development of insecticide studies, the workload of activity tests has increased sharply. Tests employing insect cell cultures combined with HTS procedures to rapidly screen insecticide candidates have been the focus of researchers in recent years. Currently, the most commonly used HTS model has been built by combining insect cell lines with the thiazolyl blue tetrazolium bromide (MTT) assay, which is utilized to determine the survival and proliferation of insect cells. This HTS model has become widely available for natural compound purification guided by activity and synthetic compound activity evaluation. For instance, the two insect cell lines AW1 and SL2 were applied as activity screening models for discovering the lead compound among synthetic pyrazole carboxamides and furanone analogs, respectively [34,35].

**Figure 2:**
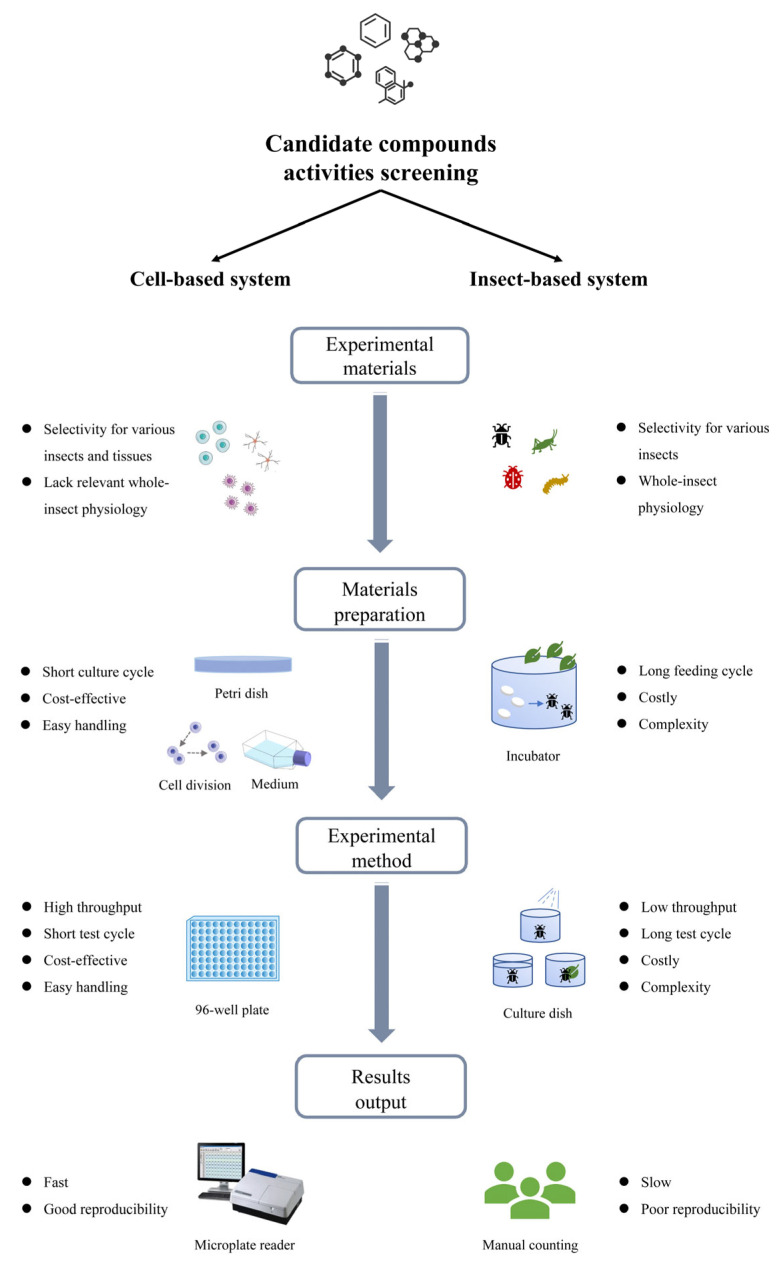
The flow chart of insecticide candidate activity screening based on cells and insects, respectively.

As summarized in Figure 2, compared to conventional insecticide toxicity tests based on insects in vivo, cytotoxic assays have notable advantages in terms of work flow. For example, as concerns rapidity for test cycles, in vitro testing has time superiority in material preparation. Compared to the time cost for feeding or hatching insects, cells may only need a couple of days for the creation of a new generation. Hence, from the overall process, in vitro testing saves time for whole test cycle. There is also a low cost for experimental materials, and feeding or hatching insects requires more manpower and material resources than cell culture. The latter may pose more expenses in terms of the initial input of the laboratory, but it has higher comparative economic benefits. In light of the results readout, in vitro testing may have more accuracy, sensitivity and easily controlled conditions. There are some new technologies emerging linked to the readout method such as infrared beam break and video recording/analysis, partly replacing traditional manual counting analysis. However, the novel method may not be available for all insect types. Especially for those insects which “feign death” or unmovable insects (scale insect), researchers often need to touch to check their status. Hence, at least for the moment, manual counting may still be the primary method for calculating the death rate of insects in vivo. In contrast, most in vitro testing only uses one colorimetric method for all cell types.

Although cell-based models have more advantages than traditional evaluation methods, much more attention should be given to reliable intra-laboratory and inter-laboratory reproducibility, high predictive power for correct toxicity assessment decisions and toxicity relevance between in vitro and in vivo models in the future (Section 4.2). In vitro models achieve higher throughput, but may not always reflect the truth. There are a number of false positive compounds that are active in vitro, but have poor biological activity in vivo. Consequently, it is hard to obtain the real active compounds by merely depending on in vitro testing. The cell-based models could not completely replace the conventional test. It may often be used as the first screening, then the many “positive hits” can be confirmed again using insect-based models.

### 3.3. Cell Death Modalities and Signaling Pathways

Insect cell-based models not only allow for efficient and accurate candidate screening, but also provide new tools and methods to uncover the mode of action of insecticidal substances at the cellular level. Active insecticidal substances can morphologically and biochemically disturb the normal vital movement of cells, eventually leading to cell death. The common modalities of cell death, include necrosis, apoptosis and autophagy, differ according to the stimuli and signal transduction. Defining cell death modalities and their relevant signaling pathways will contribute to discovering the targets (gene, protein, factor, etc.) of insecticides. Hence, there are a great number of researchers focusing on insecticides’ mode of action at the cellular level, both at home and abroad.

#### 3.3.1. Necrosis

**Figure 3:**
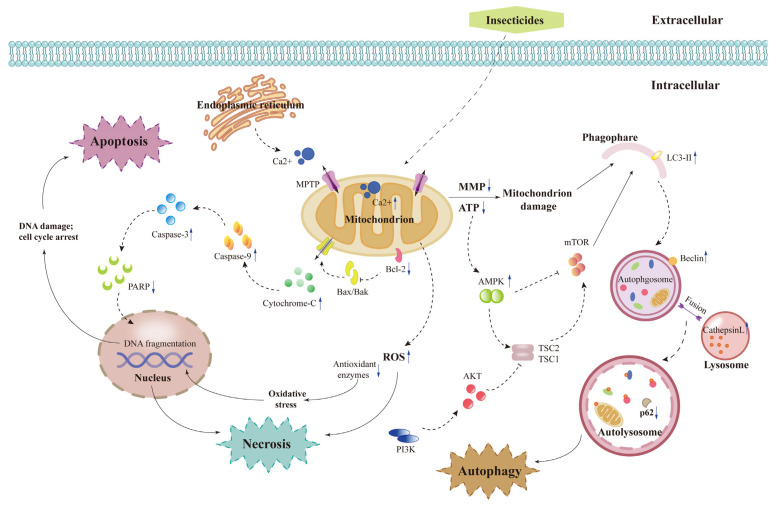
Potential cell death mode and signal pathway in insect cell lines after insecticide treatment. Insecticides could either induce apoptosis via the mitochondrial pathway, induce cell cycle arrest as a result of DNA damage, induce autophagy via the mTOR pathway or induce necrosis in insect cells. These actions of insecticides both interact with each other and work in coordination with inhibition of cell proliferation. Blue solid arrows pointing upwards or downwards represent increase or decrease in the respective substances. Black dotted arrows represent the possible routes, and black solid arrows represent the effect or action of insecticides.

Necrosis is a normal event in injured cells and is a response to external impulses including physical and chemical factors. Recent studies indicated that necrosis is an accidental or uncontrolled type of cell death accompanied by a series of cellular and molecular events. Many pesticides such as methomyl can lead to insect cell necrosis, inducing DNA damage including micronuclei, chromosome aberrations and sister chromatid exchanges in S2 cells [36]. Rotenone-induced cell death of Sf9 and SL-1 cells was shown to be accompanied by typical necrotic characteristics including plasma membrane collapse and organelle lysis, and irregular DNA degradation, instead of the typical apoptotic feature of DNA laddering [37] (Figure 3).

#### 3.3.2. Apoptosis

Apoptosis is a particular kind of PCD that can be triggered by multiple stimuli and controlled by the gene network [38]. Apoptosis is an essential biological process both in mammals and insects. In particular, the significance of apoptosis in insect growth and development has lately drawn attention to its utilization in pest management. The most representative research is the apoptosis induced by natural product pesticides. For instance, neochamaejasmin A and isochamaejasmin extracted from *Stellera chamaejasme* induced apoptosis in Sf9 and AW1 insect cells, respectively [33,39]. Proof of apoptosis was confirmed by morphological alterations and the activation of caspase-3/9. Spinosad induced mitochondrial dysfunction through strengthened reactive oxygen species (ROS) production, mitochondrial permeability transition pore (mPTP) opening and mitochondrial membrane potential (MMP) collapse, eventually resulting in cytochrome c release and apoptosis in Sf9 cells [29]. Moreover, oxidative stress and DNA damage were also observed in Sf9 cells after spinosad treatment [40]. Azadirachtin can induce serious apoptosis in Sf9 and SL-1 cells with early apoptotic features [41]. Camptothecin is not only an anticancer compound, but also a well-known insect growth regulation substance. It induced apoptosis in Sf9 cells, which showed DNA laddering and typical morphological changes including cell shrinkage and chromatin condensation [42]. Intriguingly, azadirachtin and camptothecin were involved in different apoptotic signaling pathways and/or approaches, as they caused apoptosis on different time scales. Cell cycle arrest may be an event that accompanies apoptosis. Neochamaejasmin A-treated Sf9 cells were arrested at the G2 phase, accompanied by marked DNA damage [39]. SL-1 cells were arrested at the G1 and G2 phases to repair RNAi-induced DNA damage, and the unrepaired damage led to cellular apoptosis after treatment with azadirachtin [30] (Table 1).

Researchers have shown that apoptosis in Lepidoptera is very similar to that in mammals, which can be induced through two main pathways that are recognized as the death receptor-dependent and mitochondrial-dependent pathways [43]. In the second pathway, as a response to exogenous and endogenous factors, cytochrome c is released because of mitochondrial outer membrane permeability. In the event of ATP, released cytochrome c triggers the Apaf-1 and its caspase recruitment domain, promoting apoptotic body formation. The apoptotic body is composed of procaspase-9, Apaf-1 and cytochrome c. Then, activated caspase 9 causes a cascade reaction of apoptosis effector caspase 3, which ultimately results in cell death [44]. In addition to cytochrome c, another very significant early characteristic of the mitochondrial-dependent pathway is the loss of mitochondrial membrane potential (MMP) (Figure 3). Therefore, mitochondrial outer membrane permeabilization has been suggested to take part in the mitochondrial apoptotic cascade [45]. Ren [33,34] reported that biflavones induced apoptosis in insect cells via a mitochondrial-dependent intrinsic apoptotic pathway accompanied by upregulation of cytochrome c and a proapoptotic protein Bax, and downregulation of MMP and an antiapoptotic protein Bcl-2. However, Gu [46] confirmed that the biflavone analog neochamaejasmin B induced apoptosis in AW1 cells via a death receptor-dependent pathway associated with the activation of caspase-10/8. Additionally, the chirality of biflavones might be the main reason for the above result.

**Table 1:** The cases of insect cell-based models applied to insecticide cytotoxicology research.

Organism	Cell Line	Compounds	Category	Year	Main Results and Conclusions	References
*Spodoptera frugiperda*	Sf9	Vip3Aa	Microbial insecticide	2022	Prohibitin 2 (PHB2) is a possible vegetative insecticidal protein (Vip3Aa) binding receptor detected in the membrane of Sf9 cells. PHB2 acts as an interacting partner to facilitate the internalization of Vip3Aa into Sf9 cells and keeps the stability of mitochondria	[47]
	Sf9	Avermectin	Microbial insecticide	2021	Avermectin suppresses the activity of Sf9 cells and causes programmed cell death associate with DNA damage	[48]
	Sf9	Extracts of *Cupressus macrocarpa* and *Alpinia officinarum*	Botanical insecticide	2021	MTT assay displays that golden piller is more toxic than galangal in cell viability. Both plant extracts considerably decrease the gene expression levels of chitinase and fibroblast growth factor receptor	[49]
	Sf9	Bifenthrin	Synthetic insecticide	2021	Bifenthrin substantially impacts the viability of Sf9 cells by inducing DNA damage and autophagy	[50]
	Sf9	Rotenone	Botanical insecticide	2021	Rotenone induces necrosis but not apoptosis in insect cells through a mitochondrial- and plasmic membrane-dependent pattern	[37]
	Sf9	Vip3Aa	Microbial insecticide	2020	Vip3Aa facilitates Sf9 cell apoptosis through mitochondrial dysfunction	[51]
	Sf9	Matrine derivatives	Synthetic insecticide	2020	Two recently combined compounds show finer inhibitory effect on Sf9 cell than that of the parent matrine	[52]
	Sf9	Aegerolysin-based protein complexes	Microbial insecticide	2020	The complexes permeabilise artificial lipid vesicles through aegerolysin binding to an insect-specific sphingolipid, ceramide phosphoethanolamine (CPE), and they are cytotoxic for the Sf9 insect cell line	[53]
	Sf9	1′S-1′-acetoxychavicol acetate (ACA)	Botanical insecticide	2020	ACA distills from *Alpinia galangal*. It shows higher toxicity for Sf9 cells compared to azadirachtin and it is 38-fold less toxic for HepG2 cells.	[54]
	Sf9	AcMNPV	Virus	2019	AcMNPV-Ac34-EGFP virus upregulates the progeny virus production and activates apoptosis via activation of the JNK pathway in Sf9 cells	[55]
	Sf9	β-carboline	Botanical insecticide	2019	Natural beta-carboline alkaloids control the PI3K/Akt/mTOR pathway and cause autophagy in insect Sf9 cells	[56]
	Sf9	Neochamaejasmin A	Botanical insecticide	2019	Neochamaejasmin A efficaciously causees apoptosis in Sf9 cells through mitochondrial pathways	[39]
	Sf9	β-asarone	Botanical insecticide	2019	Apoptosis induction may be one mechanism through which beta-asarone hinders the proliferation of insect cells and, thus, wields insecticidal effects	[57]
	Sf9	Harmine	Botanical insecticide	2019	Harmine causes apoptosis via extensive activation of the mitochondrial and lysosomal pathways and inhibition of DNA topoisomerase I activity in Sf9 cells	[58]
	Sf9	Curcumin	Botanical insecticide	2018	Autophagy is caused in Sf9 insect cells by curcumin via the PI3K/AKT/TOR pathway	[59]
	Sf9	Cry1Ac	Toxin	2018	Cry1Ac acts against three cell lines in terms of LC_50_ in the midgut (31.0 g/mL), fat body (59.0 g/mL) and Sf9 cell (99.6 g/mL)	[60]
	Sf9	NaF	Fluoride	2018	Sf9 cells display signs of NaF-mediated toxicity through alterations in cell morphology, apoptosis rates and protein expression	[61]
	Sf9	[1-(2-naphthyl)-3-(2-thioxo-1,3,4-oxadiazol-5-yl) beta-carbolinel (ZC-14)	Synthetic insecticide	2018	ZC-14 has a greater cytotoxicity than harmine against Sf9 cells. In addition, it displays a proliferation-resistant function in Sf9 cells via inducing apoptosis in which the mitochondrial apoptotic pathway makes a significant impact	[62]
	Sf9	Spinosad	Microbial insecticide	2018	Spinosad efficaciously provokes oxidative stress and DNA damage in Sf9 cells	[40]
	Sf9	Curcumin	Botanical insecticide	2017	Curcumin induces autophagic cell death in the Sf9 insect cell line	[63]
	Sf9	Azadirachtin	Botanical insecticide	2017	Azadirachtin could induce a considerable increase in intracellular Ca^2+^ release in the Sf9 cell line	[41]
	Sf9	Spinosad	Microbial insecticide	2017	Under ATP depletion conditions, spinosad causes autophagy of Sf9 cells and activation of the AMPK/mTOR signaling pathway	[29]
	Sf9	Fumonisin B1 (FB1)	Toxin	2017	FB1 restrains Sf9 cellular proliferation and stops cell growth at the G2/M phase	[64]
	Sf9	Cantharidin	Toxin	2017	Cantharidin causes apoptosis of Sf9 cells through the mitochondrial pathway. Bax might be necessary, but not solely for the apoptosis caused by cantharidin, and the attribution of these channels seems to be more complex	[65]
	Sf9	Extracts of *Pyrethrum* (PY)	Botanical insecticide	2017	PY hinders the viability of Sf9 cells in both a concentration- and time-dependent manner. PY could cause autophagy in the non-nervous system of insects, which may contribute to its insecticidal mechanism	[66]
	Sf9	Cry1A	Toxin	2016	An ABC transporter gene, SeABCC2b, from *S. exigua* mediates Cry1Ac cytotoxicity and, in conjunction with SeCad1b, conduces to enhanced Cry1Ca toxicity in Sf9 cells	[67]
	Sf9	Staurosporine (STS)	Microbial insecticide	2016	STS causes caspase-3 activation and apoptosis in Sf9 cells through mitochondrial-dependent inherent pathway	[68]
	Sf9	AcMNPV-BmK IT	Virus	2016	SfP53 and F-actin are the targets of viral pesticide AcMNPV-BmK IT (P10/PH) in host Sf9 cells	[69]
	Sf9	Azadirachtin	Botanical insecticide	2015	Azadirachtin causes apoptosis of sf9 cells through caspase-dependent pathways	[70]
	Sf9	Azadirachtin	Botanical insecticide	2013	Mitochondria plays a crucial role in insect cell line apoptosis	[42]
	Sf9	Fipronil	Synthetic insecticide	2013	Fipronil is cytotoxic for Sf9 cells in a time-and concentration-dependent manner	[71]
	Sf9	Rhodojaponin-III	Botanical insecticide	2011	A definite linkage for change in [Ca^2+^]_i_, cell cycle arrest and proliferation inhibition in Sf9 cells is caused by Rhodojaponin-III	[72]
	Sf9	Methoxyfenozide and methoprene	Synthetic insecticide	2011	Methoxyfenozide is more toxic than methoprene in cell viability tests. Cell growth occurs in the G2/M phase after a methoprene treatment and more modestly in G1 after methoxyfenozide treatment	[73]
	Sf9	Harmine derivatives	Synthetic insecticide	2010	The results of the integration of a series of 1,3-substituted beta-carboline derivatives exhibit that compound 2 and compound 13 are the best potential compounds, with Sf9 cells inhibition rates of 71.55% and 60.21% after 24 h treatment at concentrations of 50–200 mg/L, respectively	[74]
	Sf21	Endosulfan	Synthetic insecticide	2013	Endosulfan causes a more pronounced decrease in insect cell proliferation in comparison with mammalian cell cultures	[75]
	Sf21	Brusatol	Botanical insecticide	2013	Apoptotic death with the mitochondrial-dependent pathway caused by brusatol in Sf21 cell lines	[28]
	Sf21	Chlorpyrifos	Synthetic insecticide	2012	Sf21 cells are the most vulnerable to chlorpyrifos. Additionally, chlorpyrifos treatment causes a species-dependent reduction in cell proliferation and cell membrane damage	[76]
	Sf21	Bendiocarb	Synthetic insecticide	2012	Cells of insect origin (Sf21) are the most vulnerable to bendiocarb with significant inhibition of their proliferative activity	[77]
	Sf21	Camptothecin (CPT), hydroxycamptothecin (HCPT)	Synthetic insecticide	2012	CPT and HCPT show strong cytotoxic effects on the tested insect cell lines in a time- and dose-dependent manner	[78]
	BTI-Tn-5B1-4	Fipronil derivatives	Synthetic insecticide	2019	Several compounds have likely cytotoxicity for Hi-5 cells, particularly a 4-ethyl-substituted alkynyl Schiff base derivative (3f) that was shown to possess larger selective toxicity for the Hi-5 cell than the SL cell. In addition, 3f shows equivalent toxic activity to commercial fipronil in a Hi-5 cell, and a little toxic effect on an SL cell	[79]
*Trichoplusia ni*	BTI-Tn-5B1-4	Chlorine dioxide	Chloride	2015	The cells treated with ClO_2_ produce ROS. The produced ROS amount increases with an increase in the treated ClO_2_ amount. However, the addition of an antioxidant, vitamin E, considerably mitigates the cytotoxicity of ClO_2_ in a dose-dependent manner	[80]
	BTI-Tn-5B1-4	Coumaronochromone and flavonoids	Botanical insecticide	2012	A new coumaronochromone, 6,4’-dihydroxy-7,5’-dimethoxy-coumaronochromone, together with eleven known flavonoids is detached from the ethanol extract of the aerial part of *Derris elliptica*. All compounds indicate powerful cytotoxic activities against *Spodoptera litura* (SL) and *Trichoplusia ni* BTI-Tn-5B1-4 cells in comparison to the positive control, rotenone	[81]
	BTI-Tn-5B1-4	Azadirachtin	Botanical insecticide	2010	Azadirachtin causes BTI-Tn-5B1-4 cells’ programmed death and cytoskeletal damage	[31]
*Spodoptera exigua*	IOZCAS-Spex-II	Camptothecin (CPT), hydroxycamptothecin (HCPT)	Synthetic insecticide	2012	A considerable increase in the level of intracellular ROS accompanied by markedly increased DNA damage, lipid peroxidation and protein carbonylation after exposing to CPT and HCPT in IOZCAS-Spex-II cells	[78]
	IOZCAS-Spex-II	Camptothecin derivatives	Synthetic insecticide	2019	Compound a, synthesized by interposing 2-nitroaminoimidazoline to CPT, evidently enhances contact toxicity for the third larvae of beet armyworms, *Spodoptera exigua*, and cytotoxicity for IOZCAS-Spex-II cells	[82]
	IOZCAS-Spex-II	Pyrrolizidine alkaloids (PAs)	Botanical insecticide	2014	Compared the toxicity of PAs using both the Spodoptera exigua cell line and larval injection bioassays. Both bioassays cause similar results in the order of PA toxicity, suggest that cell lines are a useful tool for a first toxicity screening	[32]
*Drosophila melanogaster*	S2	Azadirachtin	Botanical insecticide	2016	Azadirachtin exhibits considerable cytotoxicity for S2 cells in a time- and dose-dependent manner. Azadirachtin-mediated intracellular Ca^2+^ release is the primary event that triggers apoptosis in S2 cells through both pathways of the Ca^2+^-CaM and EcR/Usp signalling cascade	[25]
	S2	Fipronil	Synthetic insecticide	2015	Fipronil effectively prompts apoptosis in S2 cells through caspase-dependent mitochondrial pathways	[83]
	SL-2	Furanone derivatives	Synthetic insecticide	2018	A total of 25 furanone analogues were used to conduct a preliminary screening on insect cell line SL2 and these compounds exhibit good cytoactivity. In particular, compound 14 presents a good inhibitory effect with an IC_50_ value of 28.14 μM	[35]
	SL-2	Furanone derivatives	Synthetic insecticide	2015	1,5-bis-(5-nitro-2-furanyl)-1,4-pentadien-3-one significantly inhibited the growth of SL2 cells. It initiated apoptosis through a mitochondrial-dependent mechanism that increased the activity of caspase-3 and altered the cell cycle	[84]
*Bombyx* *mori*	Hemocyte	Destruxin	Toxin	2014	An instant Ca^2+^ influx of hemocytes induced by destruxins A and B (DA and DB) was recorded. The DA/DB-dependent Ca^2+^ influx is not influenced by the Ca^2+^ channel inhibitors 2-aminoethoxydiphenyl borane (2-APB) and U73122. Meanwhile, an instant intracellular free H^+^ decrease caused by DA and DB is found	[85]
	BmN-SWU1	10-Hydroxycamptothecin(HCPT)	Botanical insecticide	2016	HCPT induced apoptosis via the intrinsic mitochondrial pathway in a dose- and time-dependent manner in silkworm cells	[86]
	BmN-SWU1	Hydroxycamptothecin	Botanical insecticide	2014	Bmbuffy is determined as a key homologue of Bcl-2 in silkworms.; Bmbuffy functions as an anti-apoptotic protein that interacts with Bmp53 in hydroxycamptothecin-induced apoptosis of silkworm cells	[87]
*Spodoptera litura*	SL-1	Extracts of *Torricellia tiliifolia*	Botanical insecticide	2018	4-hydroxy-3-methoxycinnamaldehyde, 3,5-dimethoxy-4-hydroxycinnamaldehyde and syringaresinol inhibit SL-1 cell survival by inducing apoptosis in both dose- and time-dependent manners	[88]
	SL-1	Extracts of *Myrsine stolonifera*	Botanical insecticide	2017	From the extracts of *M. stolonifera*, quercetin-3-O-glu-rha-glu and kaempferol-3-O-glu-rha-glu display comparable toxicities to rotenone in *M. domestica* and also exhibit cytotoxic effects on SL-1 cells	[89]
	SL-1	Azadirachtin	Botanical insecticide	2016	Azadirachtin mainly induces autophagy in SL-1 cells by dysregulating InR-and PI3K/AKT/TOR pathways, and then stimulats apoptosis by activating tAtg5	[90]
	SL-1	Extracts of *Aralia armata*	Botanical insecticide	2016	A total of 5 triterpenoids compounds affect SL-1 cell proliferation. Among them, compound 3 shows more clear proliferation inhibition activities on SL-1 cell than the positive control, rotenone	[91]
	SL-1	Extracts of *Momordica charantia*	Synthetic insecticide	2015	Momordicin I and II considerably inhibit SL-1 cell proliferation. Additionally, these compounds show suppression of cytoskeletal function, interference of mitotic figures and destruction of nuclear structure	[92]
	SL-1	Camptothecin	Botanical insecticide	2014	Programmed cell death protein 11 (pcdp 11) is upregulated in a time-dependent manner in SL-1 cells after treatment with camptothecin	[93]
	SL-1	Azadirachtin	Botanical insecticide	2012	The study revealed differentially expressed genes responsive to azadirachtin A (Aza) in *Spodoptera litura* cell line through suppression subtractive hybridization.	[94]
	SL-1	AfMNPV	Virus	2012	Cytochrome c plays an important role in apoptotic signaling pathways in Lepidopteran insect cells	[95]
	SL-1	Azadirachtin	Botanical insecticide	2011	Apoptosis induction and cell proliferation inhibition when the insect cultured cells are treated with azadirachtin A. Additionally, p53 protein is included in cell cycle arrest	[30]
	SL-HP	Cry1Ac and Cry1Ca	Toxin	2021	Insect cell lines (Hi5, SL-HP and Sf9) are susceptible to activated Cry1Ca toxin, but only SL-HP cells are also vulnerable to activated Cry1Ac toxin. Cry toxins induce autophagy in the vulnerable cell lines as shown by the analysis of the changes in the ratio of Atg8-PE to Atg8 and by formation of autophagosome dots including Atg8-PE	[96]
	SL-HP	Cry1Ac	Toxin	2016	The noncadherin-expressing Sl-HP cells are more vulnerable to activated Cry1Ac than the cadherin-expressing Hi5 cells	[97]
	SL-HP	AcMNPV	Virus	2012	Some permissive insect cells may protect against baculovirus infection via apoptosis under starvation and apoptosis is independent of the cleavage of Atg6 in SL-HP cells	[98]
	SL	Extracts of *Cacalia tangutica*	Botanical insecticide	2009	Two active ingredients, friedelin and stigmastero extract from dissimilar parts of *Cacalia tangutica*. The cytotoxicity of stigmasterol for *S. litura* cells is significantly greater than that of either friedelin or rotenone.	[99]
*Helicoverpa zea*	AW1	Neochamaejasmin B	Botanical insecticide	2022	Neochamaejasmin B curbs cell increase and it is cytotoxic to AW1 cells in a dose-dependent manner. Additionally, neochamaejasmin B induces apoptosis in AW1 cells through the caspase-10-dependent mechanism	[46]
	AW1	Biflavones	Botanical insecticide	2021	Biflavones from *Stellera chamaejasme* exhibit substantial blocking effects on Kv of AW1 cells and inhibit cell proliferation	[100]
	AW1	Isochamaejasmin	Botanical insecticide	2021	Isochamaejasmin could cause DNA damage and induce apoptosis via the mitochondrial pathway in AW1 cells.	[33]
	AW1	Lauric acid	Fatty acids	2019	Lauric acid shows the best bioactivity both in vivo and in vitro among nine FAs. Lauric acid induces apoptosis in the AW1 cells, involving the ROS levels	[34]
	AW1	Pyrazole derivatives	Synthetic insecticide	2018	Novel pyrazole carboxamides compounds exhibit good cytoactivity against AW1 cells. Among them, b5 causes AW1 cell apoptosis with a decrease in the mitochondrial membrane potential, as well as a substantial increase in the intracellular calcium ion concentration and caspase-3 activity	[101]

Supported by lots of study results, the apoptotic theory for insecticide cytotoxicology was formed. However, it is difficult to overcome the problem concerning the detection of specific targets, such as certain kinds of proteins, following identifying apoptosis bioprocesses and pathways. Thus, clarifying the key protein targets of apoptosis inducers would promote the discovery of new sites and modes of action of insecticides.

#### 3.3.3. Autophagy

Autophagy, or type II PCD, is a major stylized process for stressed cell elimination and prevention of the cytosolic proteins and organelles degrading in a highly conserved catabolic pathway [102]. Autophagy plays key roles in lots of developmental and physiological processes, such as cell death, cell survival, innate immunity and metabolism. Autophagic events, sometimes in the company of apoptosis, occur broadly in holometabolous insects, such as silkworms, honeybees and fruit flies, to eliminate organs and tissues during metamorphosis [103,104]. However, normal cell death is also caused by excessive autophagy stimulated by xenobiotics, including insecticides.

Avermectin is widely used as an internal pesticide against piercing sucking insects. Li [48] reported that avermectin showed cytotoxicity for Sf9 cells involving DNA damage and programmed cell death. Avermectin upregulates autophagy-related protein expression, including Beclin1 and LC3-II, and downregulates p62 expression. The LC3 protein is considered a hallmark of autophagy and it exists in two types, LC3-I and LC3-II. When autophagy occurs, LC3 is recruited to autophagosomal membranes and conversed from the LC3-I type to LC3-II. Beclin1 is necessary for the autophagosome membranes’ nucleation. Its connection to the preautophagosomal structure makes the Beclin1 protein function crucially in autophagy beginning and development [105]. P62 includes one LC3-interacting domain and one ubiquitin-binding domain. By linking the autophagic system and ubiquitin substrates, p62 works as the selective receptor for ubiquitin substrate degrading in autolysosomes. P62 is degraded as well when autophagy begins [106].

Harmine and harmol [58,74] showed strong autophagy induction activity. After treatment with these compounds, numerous autophagy-related genes (Atgs) increased at the RNA level, and the protein expression of Sf-Atg8 was also confirmed to be upregulated. The autophagy process starts at the phagophore assembly site: the isolated membrane expands from the phagophore into double-membrane vesicles, which are known as autophagosomes. After being recognized by and fusing with the lysosome, the forming autolysosome starts to degrade its contents [107]. This complicated procedure is regulated by many autophagy-related genes and proteins. Atg8 is the principal factor for autophagic membrane formation. Harmine sharply upregulates Atg8 in RNA and the protein level. Additionally, the other significant genes are also enhanced to complete the autophagy process, such as Atg13 and Atg101, which are associated with UNC-51-like kinase 1 and FAK family kinase-interacting protein FIP200. This complex connects to the mTOR complex 1 and negative autophagy regulator, and initiates autophagy [108].

Mammalian target of rapamycin (mTOR) acts as the major controller of autophagy responding to environmental stress. Cui [56] indicated that the PI3K/Akt/mTOR pathway mainly regulates harmine-induced autophagy in insect cells. PI3K is the intracellular signal transducer-related enzyme that causes the activation of the PI3K-Akt-mTOR pathway. PI3K could be activated by G protein-coupled receptors or growth factor receptors. Following this, in the plasma membrane, PI3K phosphorylates Akt. However, this process could be inhibited by PTEN, a kind of 3′-phosphoinositide phosphatase. Akt further triggers mTOR complex 1 via inhibition of the downstream TSC1/TSC2 complex. Phosphorylation of TSC2 by Akt results in the disruption of its complex with TSC1 and leads to mTOR activation [109]. Therefore, PI3K/Akt/mTOR is a common signaling pathway in insect cell autophagy. Li [48] and Yang [29] revealed that avermectin and spinosad could both induce autophagy in Sf9 cells through the AMPK/mTOR-mediated pathway. After treatment with avermectin and spinosad, the phosphorylated AMP-activated protein kinase (AMPK) protein increased steadily, while the phosphorylated mTOR and p70s6k protein decreased gradually in Sf9 cells. AMPK plays a significant role in energy homeostasis and can be triggered by ATP depletion, which serves as an inhibitor of mTOR. P70S6K is the downstream effector protein of the mTOR pathway. It plays a central role in cell growth and development including protein synthesis and cell proliferation. The autophagy process in insect cells is tightly and intricately regulated by a series of signaling molecules, among which a number of signal transduction pathways involve mTOR (Figure 3). This shows that mTOR acts as the core point in insect cell autophagy and may provide a new design strategy for insecticides based on the mTOR protein structure.

### 3.4. Ion Channels

Most popular commercial insecticides, whether natural products or synthetic compounds, act on relatively few targets. Among them, the ion channels in the insect nervous system represent the most efficient targets for a great majority of insecticides [110]. Ion channels are pore-forming membrane proteins that allow the flow of ions down their electrochemical gradient from one side of the membrane to the other. They form a very diverse group of proteins found in the cell membrane and in the membrane of intracellular compartments, including mitochondria, endoplasmic reticula and nuclei. The role of ion channels is not only their function as pores allowing the flow of ions. They are also involved in several cellular physiological processes, including nervous action potential propagation and excitability, apoptosis and cell proliferation [111]. Ion channels can be categorized through various aspects, such as their biophysical properties, and the stimulus gating character. The ion channels that are major targets for insecticides in the insect nervous system are as follows: voltage-gated sodium channels, voltage-gated potassium channels, calcium channels (rynodine receptors), chloride channels (GABA and GluCl receptors) and nicotinic acetylcholine receptors [112]. Experimental approaches based on insect cells combined with voltage- and patch-clamp techniques have assisted in our knowledge of the mode of action of neuroactive insecticides.

#### 3.4.1. Potassium Channels

Wang [113,114] studied the impact of cyhalothrin on the transient outward potassium current (*I_A_*) and delayed rectifier potassium current (*I_K_*) in isolated central neurons of *Helicoverpa armigera* using patch-clamp techniques. The outcomes demonstrated that cyhalothrin has neurotoxic effects on the nervous system through the regulation of activation potentials and inactivation state of *I_A_* channels. Through the activity of cyhalothrin, *I_K_* channels can be activated more easily, and the current amplitude can be inhibited significantly, which clarified that pyrethroid insecticides affected the nervous insensitivity of the central neurons of bollworms. Ren [100] reported that biflavones, the main active ingredients of the roots of *S. chamaejasme*, affect voltage-gated potassium channels (Kv) on insect neuronal cells (AW1 and WG2). The results confirmed that all three biflavones can significantly inhibit the *I_A_* compared with the *I_K_* current; among them, isochamaejasmin A stood out as having the strongest inhibitory activity against *I_A_*, with an IC_50_ value of 106.75 μM. Multiple results suggest that the inhibition of potassium current was related to the gating modification of biflavones. Isochamaejasmin A produced concentration-dependent hyperpolarizing shifts in the voltage dependence of channel steady-state activation and inactivation. 20-hydroxyecdysone showed inhibition of Sua1B cell (Anopheles gambiae) growth with no change in cellular morphology. The results showed that, in the presence of 20-hydroxyecdysone (20-HE), Sua1B cells expressed voltage-sensitive potassium channels (Kv2), and the 20-HE effect was enhanced in the presence of the potassium channel blocker 4-aminopyridine, that suggesting 20-HE played a role in cell death [115].

#### 3.4.2. Sodium Channels

Patch-clamp analyses of cockroach dorsal unpaired median (DUM) neurons have exhibited that DCJW, a decarbomethoxylated derivative, could block voltage-sensitive sodium channels in the insect. DCJW causes a dose-related restraint of the peak inward sodium current. The activity of DCJW does not affect sodium channel activation and shows no effect on either reversal potential or voltage dependence of both steady-state inactivation and sodium conductance [116]. In addition, studies on neurons of olfactory receptors in the antennae of bees showed that permethrin induced a tail current with slow attenuation in the sodium current, which showed that the two compounds bind to the sodium channel and make it difficult for the activated channel to deactivate and return to the closed state. In addition, the opening of channels can further promote the combination of compounds and channels and open most channels in the silent state. However, increase in late current indicates that the pyrethroid extends the open time of channels to delay channel deactivation [117].

#### 3.4.3. Chloride Channels

Research on native GABARs on *Drosophila* cell lines has proven that fipronil works specifically on ionotropic GABARs, while current studies on cockroach neurons have proved that fipronil also acts on GluCls. These novel outcomes suggest that GluCls and ionotropic GABARs may share certain parallels in binding sites for fipronil [118]. The known insecticidal compounds lindane and DIDS inhibit Sua1B cell growth at micromolar concentrations. Patch-clamp studies indicated that DIDS produced partial inhibition of chloride current amplitudes. In contrast, lindane increased chloride current amplitude. This finding is not only the first report of type 2 pyrethroid inhibition of insect voltage-sensitive chloride channels, but also shows that Sua1B cells express native insect ion channels with potential utility for insecticide screening [119]. The RML12 cell line derived from Aedes albopictus displays spontaneous electrophysiological activity differentiated with 20-HE. The outcomes reveal that RML12 cells could be stimulated by GABAergic antagonist as well as nicotinic agonist. The finding provides new evidence of the neuron-like functionality of the 20-HE-induced differentiated cell line, and the cell model may be employed for HTS of candidate compounds in insect nervous systems [120].

#### 3.4.4. Calcium Channels

Ryanodine receptors (RyRs) are calcium channels that regulate Ca^2+^ release from intracellular stores located in the sarcoplasmic reticulum. To discover effective insecticides targeting ryanodine receptors, Liu [121] synthesized a series of novel anthranilic diamide analogs containing N-substituted phenylpyrazole. These compounds show excellent larvicidal activities against oriental armyworms and they released stored calcium ions from the endoplasmic reticulum, which indicated that they may act as potential modulators of the insect ryanodine receptor. Dimethyl disulfide (DMDS) is a plant insecticide fumigant. DMDS was shown to reduce amplitudes of both peak transient and sustained components of the total potassium current. The DMDS-induced elevation in intracellular calcium ([Ca^2+^]_i_) modulates calcium-activated potassium currents (I_KCa_) in an unexpected bell-shaped manner via intracellular calcium [122].

### 3.5. Omics

There has been outstanding development of novel theories and technologies in life sciences. A range of “omics” technologies have appeared, in particular genomics, transcriptomics, proteomics and cytomics. Traditionally, a wide spectrum of techniques such as flow cytometry, RT–PCR and Western blot have been commonly used to identify and analyze biochemical and functional alterations in cell-based models. With these conventional methodologies, only a single or a few parameters are assessed in each assay, which is time-consuming and seriously limits the full characterization of toxic-related incidents. On the contrary, omics technologies allow the simultaneous analysis of multiple parameters in the same system and offer the opportunity to perform more extensive mechanical research on the global angles involved in insecticide toxicology. Omics can further supplement phenotype-based screenings by catching system-wide molecular responses including mRNA transcripts, proteins, metabolites and other biomolecules. In return, this could help in detecting toxic potencies, elucidating mechanisms and identifying biomarkers [123,124,125].

Insects such as *D. melanogaster*, *B. mori*, *Anopheles stephensi*, *Tribolium castaneum* and *Periplaneta americana* are promising models, and a vast amount of genomic resources facilitated the utilize of omics approaches to generate predictive and mechanistic understandings of drug toxicity. Insect cells are flexible for diverse omics platforms including transcriptomics, proteomics and metabolomics [1]. For example, by comparing the genomes or proteomes of normal and pathological cells that are treated with insecticides, some specific molecules can be identified as targets for new drug designs or provide instructions for insecticide selectivity (Table 2). Therefore, it is necessary to integrate omics theory and technology with comprehensive insecticide cytotoxicology research.

Transcriptomics based on RNA sequencing (RNA-Seq) technologies is possibly the most broadly employed omics for the in vitro study of insecticide cytotoxicology. For example, after camptothecin treatment, a total of 915 and 3560 differentially expressed genes (DEGs) were detected from different dose camptothecin treated samples, respectively. Among these were genes encoding detoxification-related proteins and components of the peritrophic membrane such as mucins and cuticle proteins. Pathway enrichment analyses indicated these DEGs were involved in DNA replication, digestion, immunity, endocrine system and metabolism [133].

As a target theory-based interaction of molecules and proteins, the analysis of the proteomic profiles in insecticide cytotoxicology might provide further global insights into insecticide mechanisms for screening new specific biomarkers or targets, directly or indirectly. For example, carbendazim exposure seriously altered 266 protein expression patterns in the heads of adult bees, in which 218 proteins revealed downregulation. Remarkably, major royal jelly proteins, a crucial multifunctional protein family with irreplaceable function in maintaining the development of colonies, were suppressed considerably after carbendazim treatment [127].

This finding was confirmed in both the head and hypopharyngeal gland of nurse bees. Furthermore, visual and olfactory loss, immune functions, muscular activity, social behavior, neural and brain development, protein synthesis and modification, and metabolism-related proteins were likely inhibited by carbendazim.

Cytomics is the extensive structural and functional investigation at the individual cell level. It has been suggested as a multi-parametric analysis to study the complex and dynamic biology in cell models. However, few studies have reported the application of cytomics technology in the field of insecticide research. Cytomics will actively participate in and play a major role in insecticide cytotoxicology studies in the future. The utilization of two or more combined omics technologies would cause a more global characterization and will contribute to a better awareness of all the involved mechanisms. Along these lines, a few studies demonstrate the utilization of this integrative strategy to study insecticide cytotoxicology. For example, transcriptomic results showed 2463 upregulated and 689 downregulated genes after harmine exposure. The enriched pathways of DEGs were primarily concerned in xenobiotic and drug metabolism. In addition, proteomics analysis showed 36 upregulated and 77 downregulated proteins. However, these results revealed a nonlinear relationship with mRNA expression [137]. Thus, the interpretation and integration of data from multiple omics are difficult, as alterations in gene or protein expression and metabolite levels occur on different time scales.

Now, we are entering an era in which omics-based technologies are progressively maturating, preparing to improve understanding of the complex mechanisms of drugs. Omics offers us “big data” after drug treatment. Among the up- or downregulation of gene or protein expression, some genes, proteins or pathways involved could directly or indirectly provide target information for drugs. This information may enhance new target discovery or conduce to explain bioprocesses. However, the potential contribution of omics-based data to mechanism studies cannot be over-emphasized [138]. Despite much research, there are few studies which further focus on the functional verification of those significant changed genes and proteins, which leads to the data analysis being superficial. Hence, we emphasized that functional verification study is necessary after we obtain and analyze these data from omics information. Moreover, omics techniques in combination with other techniques such as activity-based protein profiling (ABPP) chemoproteomic platforms have been widely applied in target identification in medicinal research [139]. This has provided good references to us for the study of insecticides’ mode of action.

## 4. Challenges and Prospects

### 4.1. Establishment of Selective Targeting Cell Lines

Insect cell-based models provide rapid, precise, specific and economical methods for insecticide research. The representative insect cell lines derived from some categories or some tissues of pests have played an important role in these models. For example, cells derived from different orders, such as Lepidoptera, Diptera, Coleoptera, etc., can be used to evaluate the insecticidal spectrum of compounds to be tested. Cells derived from different tissues of insects, such as the nervous system, intestinal tract, ovary, testis, etc., can be used to evaluate and study the targeting selectivity of insecticides. However, establishing insect cell lines with a specific objective is often a slow and challenging task. Primary cell cultures might not develop into cell lines in all cases. Most of the isolated cells might only survive for several months, and these cells might not be suitable for the aim. There are extra difficulties for developing insect cell culture on growth media for specific needs. Hence, developing techniques of cell culturing is crucial for the continued establishment of selective cell lines.

### 4.2. Insecticidal Activity Differences In Vitro and In Vivo

The standardization of cultured cells, including the cell source, liquid medium and culture methods, ensures that the cells’ living conditions are closer to those of the environment in vivo. However, living organisms are complex systems, and communication of cells among various tissues supports cell growth and proliferation. Although cell co-culture techniques may establish culture conditions consistent with the in vivo environment, the cultured cells become individual and the environment is changed completely. Therefore, the active results in vitro may be completely distinct from those in vivo. In addition, there are also differences between in vivo and in vitro tests regarding the mode of drug exposure. For example, Ren [34] reported that the toxic activity of fatty acids (FAs) shows certain differences between in vivo and in vitro tests. FAs that act on the insect nervous system need to pass through the cuticle, blood barrier and perineurium of the insect. However, insect neuron AW1 cells were exposed to FAs directly; thus, an in vitro test is more sensitive than an in vivo test. In view of this situation, the relevance of drug dose and activity must be based on full consideration of the cellular environment and biological barriers.

In vitro cell-based models suited for HTS generate many ‘hits’ but lack relevant whole-organism physiology to further validate the findings. By contrast, mammalian models provide relevant in vivo data, but are not fit for HTS and hence cannot cope with validating the large number of hits generated from in vitro screening. Thus, a vital gap exists between cell-based and mammalian models, which will be the primary problem to be solved in the future.

### 4.3. The Evaluation Models and Methods

The MTT assay is the most common method and has been broadly utilized to evaluate the proliferation of insect cells. The accuracy and feasibility of MTT methods have been debatable. Some previous studies stated that the MTT technique is not always the best to assess cytotoxicity because it strictly relates to cell metabolic activity. However, when used for cytotoxicity, it needs to be related to the number of live cells. The use of MTT alone cannot prove that there is inhibition of cell division. Thus, the MTT method should be used with caution, and alternative solutions should be considered. The additional use of flow cytometry, isotope or fluorescence labelling and other methods would help to obtain comprehensive information for the study of cytoactivties.

### 4.4. Improving Comprehensive and In-Depth Insights

Insecticide cytotoxicology, the study of the mode of action of insecticides, is a complex research topic that involves a combination of multiple processes, including genetic, metabolic and immunological factors. In the past few years, the variety and complexity of cellular models used to study insecticides have increased. Nevertheless, these single-use test assays still have many limits and need further technological advances to offer a more in-depth mechanistic knowledge of the toxic mechanisms. The use of a combination of multiple measures may provide a new valuable tool and profound perspective to study the mode of action of insecticides. For instance, many studies indicate that by regulating cytoplasmic/intraorganellar ion concentrations, providing the influx/efflux of essential signaling ions, maintaining membrane potential and controlling cell volume, ion channels are essential controllers of a number of fundamental cellular processes, including apoptosis. Malignant transformation of cells facilitated by apoptosis impairment is often accompanied by alterations in ion channel expression/function. Moreover, apoptosis, which has a strictly regulated process, involves a variety of genes, proteins and metabolites. Thus, integrating cell-based cell death modalities, ion channels and omics technologies will provide a global and in-depth point of view to reveal and verify insecticide cytotoxicology.

## 5. Summary

In summary, insect cells have been used to play crucial roles in evaluating the activity of insecticidal candidates to discover their toxic mechanisms. These cell-based studies could increase efficiency and reduce the cost of insecticide studies, providing a global and in-depth perspective to reveal and verify the mode of action of insecticides. However, challenges and limits still exist, which prevent insect cells from truly reaching their full potential as insecticide toxicological models. Nevertheless, recent advances have suggested that insect cell-based models promote the development and reasonable application of pesticides and benefit pest management.

## Figures and Tables

**Table 2:** The cases of insect cell-based models applied to omics technology.

Omics	Organism	Cell	Compounds	Year	Main Results and Conclusions	
Proteomics	*Bombyx mori*	BmN cells	BmNPV	2018	A total of 4205 identified proteins, among which 4194 are on the quantitative level. During BmNPV infection, several transcription factors and epigenetically modified proteins show substantially different abundance levels. In particular, proteins with binding activity display considerable changes in their molecular functions. Disabled non-homologous end joining by BmNPV reflects irreversible breakage of DNA. Nevertheless, highly abundant superoxide dismutase suggests that the cellular defense system is constantly functional in maintaining biochemical homeostasis.	[126]
		N cells	NaF	2015	Two-dimensional electrophoresis of whole cells extracted from BmN shows that treatment with 300 mu M NaF upregulated 32 proteins and downregulated 11 proteins when compared with controls. Identification of five different proteins through MALDI-TOF MS, four of which are identified for the first time, involving two upregulated proteins (mitochondrial aldehyde dehydrogenase ALDH2 and prohibitin protein WPH) and tqo downregulated proteins (calreticulin precursor CRT and DNA supercoiling factor SCF).	[26]
		Hemocytes	Destruxin A	2014	A total of 47 differently expressed protein spots are detected and 22 proteins in 26 spots are identified. There are eight immunity-related proteins, containing three downregulated proteins (antitrypsin isoform 3, p50 protein and calreticulin precursor) and five upregulated proteins (C-type lectin 10 precursor, serine proteinase-like protein, paralytic peptide, PPO-1 and PPO-2). Four resistance- and/or stress-related proteins (arginine kinase, carboxylesterase clade H, member 1, aminoacylase and thiol peroxiredoxin) are upregulated. Ten proteins with other or unknown functions are also recorded.	[27]
	*Apis mellifera*	Head cells	Carbendazim	2021	Handling with carbendazim seriously alters 266 protein expression patterns in the heads of adults and 218 of them exhibit downregulation after carbendazim exposure. Remarkably, major royal jelly proteins, a crucial multifunctional protein family with irreplaceable function in maintaining the development of colonies, are greatly suppressed in carbendazim-treated bees. The result is checked in both the head and hypopharyngeal gland of nurse bees. Furthermore, visual and olfactory loss, immune functions, muscular activity, social behavior, neural and brain development, protein synthesis and modification and metabolism-related proteins are likely inhibited by carbendazim treatment.	[127]
	*Helicoverpa zea*	AM1 cells	Prostaglandins (PG)	2020	Significant phosphorylation changes were observed in 31 proteins, with decreases in 15, increases in 15, and one protein showgin increased or decreased phosphorylation, depending on PG treatment. Increasing PG exposure times leads to changes in fewer proteins; 20 min incubations led to changes in 16 proteins, 30 min to changes in 13, and 40 min to changes in 2 proteins. The proteins are identified using bioinformatic analyses, involving transcript description, calculated molecular weights and isoelectric points, molecular weight search score, total ion score, numbers of peptides, percent protein coverage, E-value and highest peptide score.	[128]
	*Spodoptera frugiperda*	Sf9 cells	AcMNPV mutants (lacking p35 gene)	2016	A total of 4004 sf9 proteins were identified using iTRAQ. After comparison of the substantially expressed 483 proteins from p35k0AcMNPV-infected Sf9 cells and the considerably expressed 413 proteins from wtAcMNPV-infected Sf9 cells, it was found that 226 proteins were specific to p35koAcMNPV-infected Sf9 cells. The most upregulated proteins relate to Epstein–Barr virus infection, RNA transport, calcium signaling pathway, cGMP-PKG signaling pathway, oxidative phosphorylation and N-Glycan biosynthesis.	[129]
Transcriptomics	*Aedes albopictus*	U4.4 cells	West Nile virus (WNV) and Lammi virus (LamV)	2022	WNV-infected cells have upregulation of a broad range of immune-related genes, while, in LamV-infected cells, many genes related to stress, such as various heat-shock proteins, are upregulated. The transcriptome profile of the dual-infected cells is a mix of up- and downregulated genes triggered by both viruses.	[130]
	*Drosophila melanogaster*	Ovary cells	Cyromazine	2022	Cyromazine reduces the number of germ cells by interfering with the ecdysone signaling pathway. Results indicate a significant decrease in the expression of ecdysone signaling-related genes compared to the control group. Furthermore, the titer of the ecdysone hormone is also markedly reduced (90%) in cyromazine-treated adult ovaries, suggesting that ecdysone signaling is immediately related to the decrease in the number of germline stem cells and cystoblasts.	[131]
		Kc cells	Deltamethrin (DM)	2020	Identified 268 DEGs in Kc cells treated with DM, including 180 upregulated genes and 88 downregulated genes. When the cells are treated with DM in the case of overexpression of the Keap1 gene, the cytochrome P450 family genes are considerably downregulated, and some disease-related genes and non-coding genes also are changed. The data show that the Keap1-Nrf2-ARE pathway may play an important role in DM stress.	[132]
	*Spodoptera frugiperda*	Midgut cells	Camptothecin (CPT)	2021	A total of 915 and 3560 DEGs were identified from samples treated with 1.0 and 5.0 µg/g CPT, respectively. Among the identified genes are those encoding detoxification-related proteins and components of the peritrophic membrane such as mucins and cuticle proteins. KEGG pathway enrichment analyses indicate that part of DEGs is involved in DNA replication, digestion, immunity, endocrine system and metabolism.	[133]
	*Achaea janata*	Midgut cells	Bt formulation	2019	A total of 34,612 and 41,109 transcripts were detected in controls and larval midgut samples exposed to toxins, out of which 18,836 in the control and 21,046 exposed to toxins of samples are elucidated. Microarray data analysis employed to monitor the gene expression in Cry toxin exposure revealed that 375 genes are upregulated and 579 genes are downregulated during all the time points (12–60 h) of toxin exposure. The differentially expressed transcripts contain Cry toxin receptors, gut proteases, arylphorin, REPATs, detoxification enzymes and aquaporins. Validation of microarray data is performed with real time quantitative PCR using few randomly selected genes and the results obtained are in corroboration.	[134]
	*Mamestra configurata*	Midgut cells	MacoNPV-A	2014	The earliest genes identified by each method have substantial overlap, comprising known early genes as well as genes unique to MacoNPV-A and genes of unknown function. The RNAseq data also disclose a wide variety of expression levels across all ORFs, which could not be measured using qPCR. This dataset provides a first whole genome transcriptomic analysis of viral genes required for virus infection in vivo and will provide the basis for operationally evaluating specific genes that may be critical elements in baculovirus midgut infection and host scope.	[135]
Transcriptomics and proteomics	*Spodoptera frugiperda*	Sf9 cells	Harmine	2020	Transcriptomic analysis revealed 2463 upregulated and 689 downregulated genes following harmine treatment. The most commonly enriched pathways of DEGs are mostly concerned in drug and xenobiotic metabolism. Proteomics analysis revealed 36 upregulated and 77 downregulated proteins, and the results show a non-linear relationship with mRNA expression. All the genes connected to detoxification and resistance in the transcriptome and DEGs are identified and annotated. Complete open reading frames of 27 cytochrome P450s (CYPs), 27 glutathione 5-transferases (GSTs), 11 carboxylesterases (CarEs), 10 UDP-glucuronosyltransferases (UGTs) and 29 heat shock proteins (HSPs) are gathered and verified using qRT-PCR.	[136]

## Data Availability

Data will be made available on request.
